# Addictive and other mental disorders: a call for a standardized definition of dual disorders

**DOI:** 10.1038/s41398-022-02212-5

**Published:** 2022-10-13

**Authors:** Nestor Szerman, Marta Torrens, Rafael Maldonado, Yatan Pal Singh Balhara, Caroline Salom, Icro Maremmani, Leo Sher, Javier Didia-Attas, Jun Chen, Ruben Baler, World Association on Dual Disorders (WADD)

**Affiliations:** 1 Institute of Psychiatry and Mental Health, University Hospital Gregorio Marañon, Madrid, Spain; 2 WPA Section on Dual Disorder, Geneva, Switzerland; 3 Addiction Programme- Hospital del Mar, IMIM-Hospital del Mar Medical Research Institute, Universitat Autònoma de Barcelona, Barcelona, Spain; 4 Laboratory of Neuropharmacology, Department of Experimental and Health Sciences, Universitat Pompeu Fabra, Barcelona, Spain; 5 Hospital del Mar Medical Research Institute (IMIM), Barcelona, Spain; 6 National Drug Dependence Treatment Centre (NDDTC) and Department of Psychiatry, All India Institute of Medical Sciences (AIIMS), New Delhi, India; 7 Institute for Social Science Research, The University of Queensland, Brisbane, QLD Australia; 8 National Drug & Alcohol Research Centre, UNSW, Sydney, Australia; 9 ARC Centre of Excellence for Children & Families over the Life Course, Brisbane, QLD Australia; 10 University of Pisa. UniCamillus, International Medical University in Rome, Rome, Italy; 11 James J. Peters VA Medical Center, New York, NY USA; 12 Icahn School of Medicine at Mount Sinai, New York, NY USA; 13 Columbia University College of Physicians & Surgeons, New York, NY USA; 14 Servicio de Psiquiatría y Escuela de Medicina Hospital Italiano, Buenos Aires, Argentina; 15 Office for Clinical Research Center, Shanghai Mental Health Center, Shanghai Jiao Tong University School of Medicine (SMHC), Shanghai, China; 16 Office of Science Policy and Communications, National Institute on Drug Abuse, National Institutes of Health, Bethesda, MD USA

**Keywords:** Psychiatric disorders, Neuroscience

## Abstract

The persistent difficulty in conceptualizing the relationship between addictive and other mental disorders stands out among the many challenges faced by the field of Psychiatry. The different philosophies and schools of thought about, and the sheer complexity of these highly prevalent clinical conditions make progress inherently difficult, not to mention the profusion of competing and sometimes contradictory terms that unnecessarily exacerbate the challenge. The lack of a standardized term adds confusion, fuels stigma, and contributes to a “wrong door syndrome” that captures the difficulty of not only diagnosing but also treating addictive and other mental disorders in an integrated manner. The World Association on Dual Disorders (WADD) proposes the adoption of the term “Dual Disorder” which, while still arbitrary, would help harmonize various clinical and research efforts by rallying around a single, more accurate, and less stigmatizing designation.

## Introduction

The advancement of a scientific mission relies on precise communication, and consistent messaging, including standard nomenclatures, plays a key role in that regard. Language has the power to shape people’s thoughts and beliefs: it can inspire, rally, and unite individuals toward common and positive goals, but it can also contribute to the emergence of wrong assumptions and stigmatizing stereotypes. The words we choose to describe the manifestation of an addictive disorder in association with other mental disorders offer good examples of the potentially detrimental aspects of language. Certain terms can have a significant impact on, among others, whether affected individuals will seek help or the quality of the treatment they receive. Here, we propose the term “Dual Disorder” (DD) as an apt descriptor of this clinical entity and provide the reasoning behind our recommendation of its adoption as standard nomenclature. We believe this will facilitate public and professional discourse in the field and help reduce the stigma and discrimination around psychiatric illnesses in general and addictive disorders [substance use disorders (SUD) and behavioral addictions] in particular.

## A brief history of dual disorders

Multiple epidemiological studies have established that DDs are an expectation rather than an exception: a substantial fraction of patients suffering from a mental disorder at some point in their lives will also experience an addictive disorder, and vice versa [1, 2], which depending on various demographic factors and specific disorder dyads, can range from around 40–60% [3–5]. For the uninitiated, dual disorders are to be expected when it comes to treating people with various mental disorders, a prevalence that increases as the severity of mental disorders increases. More than 75% of severe psychiatric disorders occur with other mental disorders, such as SUD and other addictions [6].

If we take the perspective of those who seek addiction treatment, although the data are quite variable, close to 70% of them will present another mental disorder [7]. These data most likely reflect an underestimate, to the extent that only diagnostic categories and not symptomatic dimensions were used in the assessment. This kind of information is significantly underrecognized among mental health experts, whether they work within a mental health or addiction care network.

The accumulated evidence suggests that dual disorders reflect etiological overlaps, common contributing factors, and bidirectional relationships between paired conditions [8, 9]. For example, according to the National Epidemiological Survey on Alcohol and Related Conditions (NESARC) study, 96% of patients suffering from pathological gambling disorder have other mental disorders, depression being one of the most frequent [10]. Importantly, 87% of these patients display high impulsivity [11], probably a key marker prevalent among those suffering from depression. Thus, depression could be a specific phenotype that occurs with some measures of impulsivity [12, 13]. Similarly, there is robust evidence to suggest that maladaptive emotion regulation (ER) is central to the development and maintenance of a broad range of psychopathologies, including SUD [14]. The mediating role of dysfunctional ER in the bidirectional relationships between SUD and suicidality [15], offers another good example of the usefulness of transdiagnostic constructs.

Importantly, dual disorders are frequently under-reported [16] and under-treated [17], partly due to the persistent challenges in understanding and classifying them [18, 19]. Their high prevalence, combined with huge diagnostic and treatment gaps, results in a significant burden for patients, their relatives, and society at large [20]. One of the most deleterious consequences of these systemic inadequacies is the so-called “wrong door syndrome”, characteristic of a persistently siloed healthcare landscape, that affects patients with dual disorders who are not sure where to find the right treatment [21]. Adoption of a DD-based nomenclature could increase clinicians’ awareness of the close and, at the same time, transdiagnostic relationships between the signs and symptoms of addictive and other mental disorders and help them address their etiologies and clinical manifestations in a more integrated and effective way [22].

The lack of consensus on standard terminology has resulted in the proliferation of competing labels, contributing to increasing societal stigma and self-stigma for affected individuals. For starters, the World Health Organization (WHO) usually refers to “dual diagnosis” when a SUD presents together with another psychiatric disorder in the same person, but it also encompasses any two psychiatric or even any two SUD [23], thus the term is nonspecific by definition. This may help explain why this organization has begun to use the term dual disorder, at least in one of its more recent reports [22]. Meanwhile, the European Monitoring Centre for Drugs and Drug Addiction (EMCDDA) has used the terms “comorbidity” [24] and “dual diagnosis” [25] interchangeably to describe the temporal coexistence of two or more psychiatric disorders, as defined by the International Classification of Diseases (ICD), when one of them is problematic substance use [26]. The American Association of Addiction Medicine (ASAM)’s criteria for patient placement also uses dual diagnosis, although interchangeably with yet another term: “co-occurring disorder” (COD) [27], which seems to also be the preferred term for the Substance Abuse and Mental Health Services Administration (SAMSHA) in the US [28]. Meanwhile, in the Spanish, French, Portuguese, and Italian languages, the most widespread and accepted term is “dual pathology” [29]. But there is more: we can find other particularly troubling (i.e., stigmatizing) terms in some older articles [30] or nonscientific literature [31] that refer to a population of patients with addictive disorders and other mental illnesses as “mentally ill chemical abusers” (MICA)(or its reverse: “chemically addicted mentally ill”). For reasons that will be explained in the next section, the WADD prefers the term “Dual Disorder” (DD), which has been introduced progressively, at least since the early 90 s [32], and already enjoys substantial support in that both, the UN Commission on Narcotic Drugs (UNCND) [22] and the World Psychiatric Association (WPA) has among its different scientific sections one on “dual disorders”, while an influential US treatment guideline has also been recommending its use for the past two decades [33].

While the existing lack of consensus about the proper nomenclature hinders both research and clinical efforts, it is just the tip of the iceberg: Underneath this cacophony of terms lie many different and often conflicting schools of thought about the nature of this complex and neglected condition. The reality is that DD have been ignored or even denied for years and that in many settings, the disorder is poorly understood or overlooked altogether.

## Using science to chart a path forward

Our field has long been saddled with a lack of clarity on whether DD represent distinct entities or alternative clinical manifestations of a single core, underlying pathophysiological process. The reality is that many patients present with a heterogeneous collection of addictive and other mental disorders, and these symptoms and their severity can change over time [34].

There is broad scientific consensus that all mental disorders, including addiction, are disorders of the brain. This consensus, while not monolithic (some authors opt for a more nuanced approach, although still neurobiological in nature when it comes to SUD [35]), is rather robust and based on multiple lines of evidence [36, 37]. Yet, despite such broad agreement, references to addictive and other psychiatric disorders as separate entities are still common, as if the former were fundamentally different from the latter. If addictions are mental disorders, it behooves us to refer to “addictive and other mental disorders”, a turn of phrase in which the order is important because it denotes that an addiction (whether drug-related or behavioral) is also a mental disorder and, therefore, a brain disorder.

Moreover, it would be difficult to argue that addictive, and other mental disorders are two completely different types of mental disorders, for this would require the highly unlikely assumption that the high degree of co-prevalence between them is the result of random factors or measurement artifacts. In fact, the NESARC study has demonstrated, for example, that purely substance-induced mood disorders (SIMD) accounted for a very small percentage of mood disorders among all those with SUD [38]. Similar patterns of comorbidity and risk factors in individuals with SIMD and those with mood depressive disorder suggest that the two conditions likely share underlying etiological factors [39]. The emerging consensus is that addictive and other mental disorders are strongly linked, albeit via complex and not necessarily direct relationships. Indeed, a range of factors is likely to contribute to the particularly strong linkage between a lifetime diagnosis of addictive and other mental disorders, with specific early-life events and factors identified as contributing more strongly to the emergence of dual compared to single disorders [40, 41]. Progress in the neurosciences is providing new perspectives from which to identify the underlying mechanisms involved in the onset and development of addictive disorders. In the case of SUD, they have spurred better pathophysiological theories [42, 43] with the power to improve our understanding of their multi-level interactions with other psychiatric disorders. Similar lines of thinking are also being applied to other addictive disorders, such as gambling or compulsive sexual behavior [44, 45].

## From molecules to environment

Neuroscience has shown that addictive and other mental disorders often display sets of interconnected and/or overlapping brain processes, rather than being disorders primarily defined by a single behavior (such as uncontrollable excessive drug use) [46]. These connections operate at multiple phenomenological levels, but the clearest examples may be the neurotransmitter systems that show deficits in various psychiatric conditions and that are also the direct targets of addictive drugs. To state the obvious, all psychoactive substances with addiction liability have a counterpart or connection with one or more endogenous systems, such as the dopaminergic, opioidergic, endocannabinoid, or cholinergic-nicotinic systems [47, 48]. Therefore, an inherited or acquired impairment in any of these neurotransmitter systems and circuits could help explain common underlying risks of suffering both addictive behaviors and other psychiatric symptoms, including pathological personality traits or disorders [29, 49]. Recent advances in our understanding of such interindividual differences reinforce the need to incorporate the drug-of-choice model [50]. This model considers that people may be more susceptible to a certain drug or class of drugs (or to compulsive video game use, for example), based on individual differences, and different mental disorders or symptoms, including endophenotypes, such as personality traits. It is well known that the administration of psychoactive substances do not have the same effects among different individuals [51, 52]. One of the clearest examples is that stimulants calm down people with Attention-Deficit/Hyperactivity Disorder (ADHD), but not others, by correcting imbalances in dopamine and norepinephrine levels [53]. This differential effect on different people/brains can be transferred to all psychoactive substances such as nicotine, alcohol, cannabis, cocaine, and opioids, as evinced by a growing scientific literature.

At the next level of analysis, research in genetics and precision psychiatry has uncovered significant evidence that some DD dyads (e.g., cannabis/attention deficit [54], tobacco use disorder/schizophrenia [55], alcoholism/depression [56], gambling/ADHD [57–59], drug use/schizophrenia [60]; smoking/suicide attempts [61], cocaine/ADHD [62], seem to show at least some common genetic bases. Such sharing of genetic underpinnings presents a towering challenge to the rigid compartmentalizing diagnostic boundaries that separate addictive from other psychiatric disorders, one with far-reaching implications for translational research and therapeutic outcomes [63].

In addition, and similar to addictive disorders, the prevalence and severity of a DD is also linked to the early onset of the disorder [29, 64], befitting the model of developmental disorders that often begin during late childhood and adolescence and may present with different phenotypes, whether addiction-related or other personality traits, such as disorganized attachment [65], impulsiveness [66], etc. Our growing understanding of the many ways in which environmental factors, such as trauma and early-life stress [67, 68] or sleep deficits [69, 70], can perturb brain development and function and increase the risk of addictive and/or other mental disorders, offers additional compelling arguments towards an improved explanation of the complex pathophysiology of DDs [71].

The combined analysis at the genetic, neurophysiological, and developmental levels, is bringing the bidirectional nature of these relationships into sharper focus. It is clear that the chronic use of any psychoactive (including addictive) substances can jeopardize various aspects of brain activity, like blood flow, neurotransmitter activity, structure, and functional connectivity, in ways that could either trigger or exacerbate the symptoms of a mental illness [9]. Additionally, it is hardly surprising to discover that unmet mental healthcare needs are closely linked to the consumption of psychoactive substances that can lead to a SUD [51, 72]. This is consistent with the self-medication hypothesis, as revisited from a neurobiological perspective [73]. And at the behavioral level, overlaid atop mechanistic arguments, is the fact that rigid or inflexible emotional responses and atypical patterns of thinking and behaving are central to many, if not all, of the emotion-related disorders, including depression, anxiety, stress, eating, substance, and some personality disorders [74].

When combined, these data points offer new insights into the many ways in which brain function can be perturbed and help explain the high prevalence of DD. Advances in this area could usher in new approaches to enable healthcare professionals to offer more adequate personalized assessments and evidence-based treatments for people with DD. Importantly, it is worth emphasizing that the benefits of adopting a standard term would extend not only to addictive disorders involving psychoactive substances but to any behavioral addiction, such as gambling disorder [75], internet gaming disorder [76], or social network site addiction [77, 78], as they move through the various stages of the clinical recognition process [79]. Behavioral addictive processes, not involving psychoactive substances, are also likely to share multiple neurobiological and genetic links with some substance use and, by extension, other psychiatric disorders. For example, brain imaging studies are consistent with the notion that both pathological gambling (DSM-5 TR) and gaming disorder (ICD-11), which are the only behavioral addictions included in internationally recognized classifications, display deficits in neuronal pathways implicated in behavioral control, which, similar to the case of SUD, display enhanced impulsivity as an underlying vulnerability [80]. On the other hand, the similarities (and differences) between SUD and other (non-substance) addictive behaviors that are under consideration for future designation as behavioral addictions, are also beginning to shed some light on the potential overlaps. One key example is a reduced ability to delay gratification, long observed in SUD, that appears to be associated with some forms of obesity [81] as well as with social media addiction [82]. Finally, the genetic, individual, and environmental factors that increase vulnerability for developing some SUD are also similar to those promoting behavioral addictions not involving substances [83, 84] and, of course, other mental disorders.

## The nomenclature dilemma

One of the obstacles in the pursuit of a more rational, neuroscience-based classification of DD (as well as other complex mental disorders more broadly) stems from the fact that DSM-based instruments are not well suited to address complex phenomena, for they use diagnostic categories (rather than symptom dimensions) that may not always reach the diagnostic criteria threshold. It is evident that, despite the advantages of having a categorical diagnostic system based on the observation of psychopathological phenomena, just as Kraepelin did in the early days of psychiatry, ca. 1915 [85], the lack of sensitivity and reliability makes it difficult to capture the broad spectrum of mental symptom manifestations and the relationships between them [86]. This helps explain why the DSM and ICD have systematically and consistently overlooked DD across successive revisions.

This lack of categorization clarity has provided fertile grounds for the proliferation of competing terms that aim to capture the same phenomenon. It is worth pointing out that every one of these terms, including DD, is arbitrary and colored by the “realist” tradition that considers constructs such as schizophrenia or SUD as true reflections of mental phenomena. This, in spite of the fact that we can only observe the signs, symptoms, and course of the illnesses we postulate result from these disorders [87]. Thus, it would be highly desirable to explore next-generation “instrumentalist” approaches that consider existing constructs as mere tools to be evaluated on their empirical adequacy [87]. It is evident that in this third decade of the 21st century, an era of breathtaking neuroscientific advances and the dawn of personalized medicine and precision psychiatry, patients and their relatives have a right to expect more than diagnoses based on the phenomenological description of their experiences [50]. Unfortunately, we are not yet in a position to deploy a new classification of mental disorders. While we wait for the many ongoing laudable efforts in this direction [88–91] to bear fruit, the WADD proposes adopting DD as the preferred standard terminology, a recommendation that is based not only on the listed drawbacks suffered by the competing terms but also on its own merits.

The term “comorbidity”, coined by Fenstein in 1970 to indicate the coexistence of two different and separate diseases [92], has been used in psychiatry when two different diagnostic categories coexist, for example, tobacco use disorder and schizophrenia, with the obvious implication that these two symptomatic expressions remained unrelated. The term “dual diagnosis” operates under a similar rationale since it refers to two categorically different diagnoses. This debate goes back many years when it was proposed that the definition of comorbidity merely specifies an association in time, not necessarily a causal relationship, between conditions [93]. Similarly, the terms “concurrent disorders,” and “co-occurring disorders” imply a purely temporal relationship, which could reflect either a common underlying cause or completely unrelated etiologies [94].

On the other hand, the term DD offers a broad, symptomatic, and dimensional view of the condition that includes different mental disorders or symptoms, including personality traits (endophenotypes), that determine vulnerability or resilience to addictive and other mental disorders [95]. The result is a more consistent heuristic framework for conducting translational research on mental disorders. The dual disorder is also the term that most naturally conveys the need for both broad assessments to identify multiple conditions as well as appropriately integrated interventions that could modify the trajectory of DD by abandoning the simplistic notion that these are brain disorders featuring different psychopathological expressions. The term dual disorders, unlike dual diagnosis or comorbidity, includes not only a unified vision of two diagnostic categories (DSM-5) but also transdiagnostic, syndromic, and symptomatic dimensions, which may be simultaneous or sequential during the life span, and that could be readily incorporated into Research Domain Criteria (RDoC) type projects, allowing us to advance more steadily towards precision psychiatry [96].

Somewhat paradoxically, a DD-based framework would also be consistent with DSM and ICD polythetic criteria whereby specific mental disorders are defined by multiple symptoms, not all of which need to be present or currently active (e.g., in a “preaddiction” stage [86] or when a SUD is in early or sustained remission) to consider a mental disorder present in a specific individual [97]. Thus, the term DD is compatible with DSM/ICD operational definitions whereby DD represent not only the temporary coexistence of different disorders but also the sequential events that manifest themselves at different points along the life span and the course of a person’s mental illness.

Finally, as suggested throughout this perspective, an equally important benefit of the term is that it could help alleviate the stigma and discrimination that adds to the suffering of patients with a dual disorder: The main competing terms (i.e., dual diagnosis, and comorbid or co-occurring conditions), imply two different diagnostic entities (hence, separate conditions) that are individually rooted in DSM categorizations and merely happen to occur in one person. We believe the proposed harmonization opens up the possibility of going beyond the diagnostic categories of the DSM, one of whose problems is not considering dual disorders, and thus include dimensions of mental symptoms and dysfunctional personality traits that could enable a more accurate diagnosis and clinical management. The concept “Dual Disorder” (one complex condition) shifts the focus to the individual patient, promoting a more personalized approach because it evokes the complex nature of seemingly unrelated manifestations contributing to one condition and making it possible to treat the person and not just an addictive or other mental disorders.

## Conclusion

It is evident that consensus-building efforts are needed to facilitate the adoption of a common term to define the clinical reality of dual disorders. Here, we call for the adoption of “Dual Disorder” as the standard term in research work and clinical practice. We believe that this would constitute an important step forward not only to improve the education of health professionals but also to achieve better integration of mental health and addiction services when treating a single person suffering from different manifestations of mental disorders. Also, this new perspective must reach patients, their families, and society in general, suffering from disorders that have been stigmatized, misunderstood, discriminated against, and mishandled for too long, and making it possible for them to find “the right door” leading to effective recovery.
